# Prevalence of reproductive tract infections among women preparing to conceive in Chongqing, China: trends and risk factors

**DOI:** 10.1186/s12978-022-01502-x

**Published:** 2022-10-03

**Authors:** Jun Liu, Mengyao Zeng, Liu Yang, Yanyan Mao, Yang He, Min Li, Qing Chen, Weijin Zhou, Liang Chen, Qianxi Zhu

**Affiliations:** 1 NHC Key Laboratory of Birth Defects and Reproductive Health (Chongqing Population and Family Planning Science and Technology Research Institute), Chongqing, 400020 China; 2 NHC Key Lab. of Reproduction Regulation, Shanghai Institute for Biomedical and Pharmaceutical Technologies, Fudan University, Shanghai, 200237 China; 3 School of Public Health, Fudan University, Shanghai, China; 4 West China School of Public Health and West China Fourth Hospital, Sichuan University, Sichuan, China

**Keywords:** Reproductive tract infections, Epidemiology, Prevalence, Sexually transmitted infections, Risk factors, Endogenous infections, Trend

## Abstract

**Background:**

Reproductive tract infection has become a major public health issue all over the world for its high and growing prevalence. It can cause adverse pregnancy outcomes in pregnant women and their foetuses. This study aimed to investigate the trends and risk factors of the prevalence of reproductive tract infections among women who prepared to conceive in the Chongqing Municipality (China) from 2012 to 2016.

**Methods:**

A multi-center cross-sectional study was conducted between January 2012 and December 2016. Women aged 20–49 years who intended to get pregnant were recruited for this study. All participants underwent preconception examination, which included testing for *Neisseria gonorrhoeae*, *Chlamydia trachomatis*, *Trichomonas vaginalis*, syphilis, bacterial vaginosis and candidiasis according to the national diagnostic standard. A total of 439,372 women with testing results for all six types of reproductive tract infections were included in our final analyses. Logistic regression and factor analysis were used to determine the possible sociodemographic factors associated with prevalence trends.

**Results:**

In our study, the overall positive rate of RTIs among the 439,372 women of reproductive age was 5.03%. Candidiasis was the most common infection in our population (2.47%), followed by bacterial vaginosis (1.28%), syphilis (0.73%), *T. vaginalis* (0.49%), *C. trachomatis* (0.20%) and *N. gonorrhoeae* (0.06%). The prevalence of reproductive tract infections was highest among women aged 35 years and above, with a primary or lower education level, history of pregnancy, delivery, induced abortion, or spontaneous abortion. From 2012 to 2016, the trend of the overall prevalence of reproductive tract infections was V-shaped, decreasing steadily from 2012 to 2015, with a slight rise in 2016. Our results suggest that the distribution change of age, education level, gravidity, parity, and history of induced abortion influenced this trend.

**Conclusion:**

Since the number of high-risk women who intend to become pregnant is growing in the Chongqing Municipality, pre-conception positive preventions including health education, regular screening, and timely treatment of reproductive tract infections are needed to prevent the impact of reproductive tract infections on maternal health and infant safety.

**Supplementary Information:**

The online version contains supplementary material available at 10.1186/s12978-022-01502-x.

## Plain Language Summary

Reproductive tract infections (RTIs) can cause serious health problems, such as spontaneous abortion and congenital diseases in pregnant women and their foetuses. However, there is a lack of studies focusing on the prevalence of RTIs and their trends among women who intend to conceive. This cross-sectional study examined data collected from 439,372 women during their preconception care to investigate the trend and related risk factors of the prevalence of RTIs, thereby providing essential data for their prevention. Participants were women from all 39 counties in the Chongqing Municipality of China, and data were collected between 2012 and 2016. We found that the overall prevalence of the six types of RTIs among these women was 5.03%, which was relatively lower than that in other populations in previous studies. Age, education level, history of pregnancy or delivery, and history of abortion were all associated with the prevalence of RTIs. The prevalence trend from 2012 to 2016 was V-shaped decreased steadily from 2012 until 2015 and rose slightly in 2016. Our data suggested that this trend might be influenced by changes in the proportion of ‘high-risk’ women, that is, women with higher age, lower education level, and a history of pregnancy or induced abortion. This study suggests that health education and regular screening are necessary to face new challenges experienced by older women or women with previous pregnancies who intended to get pregnant in recent years in China.

**Supplementary Information:**

The online version contains supplementary material available at 10.1186/s12978-022-01502-x.

## Background

Reproductive tract infections (RTIs) are infections of the genital tract caused by bacteria, fungi, viruses and other pathogens [1]. According to the latest report of the World Health Organization (WHO), in 2016, the estimated global incident cases of four types of RTIs (chlamydia, gonorrhoea, trichomoniasis, and syphilis) was 376.4 million, with an increasing trend compared to 2012. Furthermore, the majority of RTI patients were in developing regions [2]. According to the 2006 National Population and Family Planning Sample Survey in China, the prevalence of RTI reached 34.1% among women aged 15–49 years [3]. Moreover, several population-based studies showed that RTIs were more common among married women living in rural areas [4–7].

Although RTIs can affect both men and women, the consequences for women, especially for pregnant women, are more common as well as more severe. RTIs can cause serious health problems for both mothers and their foetuses. These include spontaneous abortion, stillbirth, congenital disease, and puerperal sepsis [8, 9]. Due to their heavy socio-economic burden, RTIs have become a major public health issue all over the world. For this reason, clear understanding, effective screening, and targeted strategies are necessary to prevent the harmful effects of RTIs in women who prepare to conceive. Although previous studies have focused on the prevalence of RTIs and their risk factors in China, findings varied according to local cultural customs, economic status, and demographic composition [6, 10, 11]. In addition, with the development of technology, diagnostic methods varied according to time and location of the study [12, 13]. Moreover, few studies have investigated the prevalence of RTIs among women who prepared to conceive in recent years.

To provide data support for the prevention of RTIs among women who intended to become pregnant in the Chongqing Municipality of China, we conducted this population-based cross-sectional study, which covered all 39 counties of Chongqing. The study aimed to investigate the trend and risk factors of RTIs among women aged 20–49 years preparing to conceive between 2012 and 2016.

## Methods

### Study design and participants

**Fig. 1:**
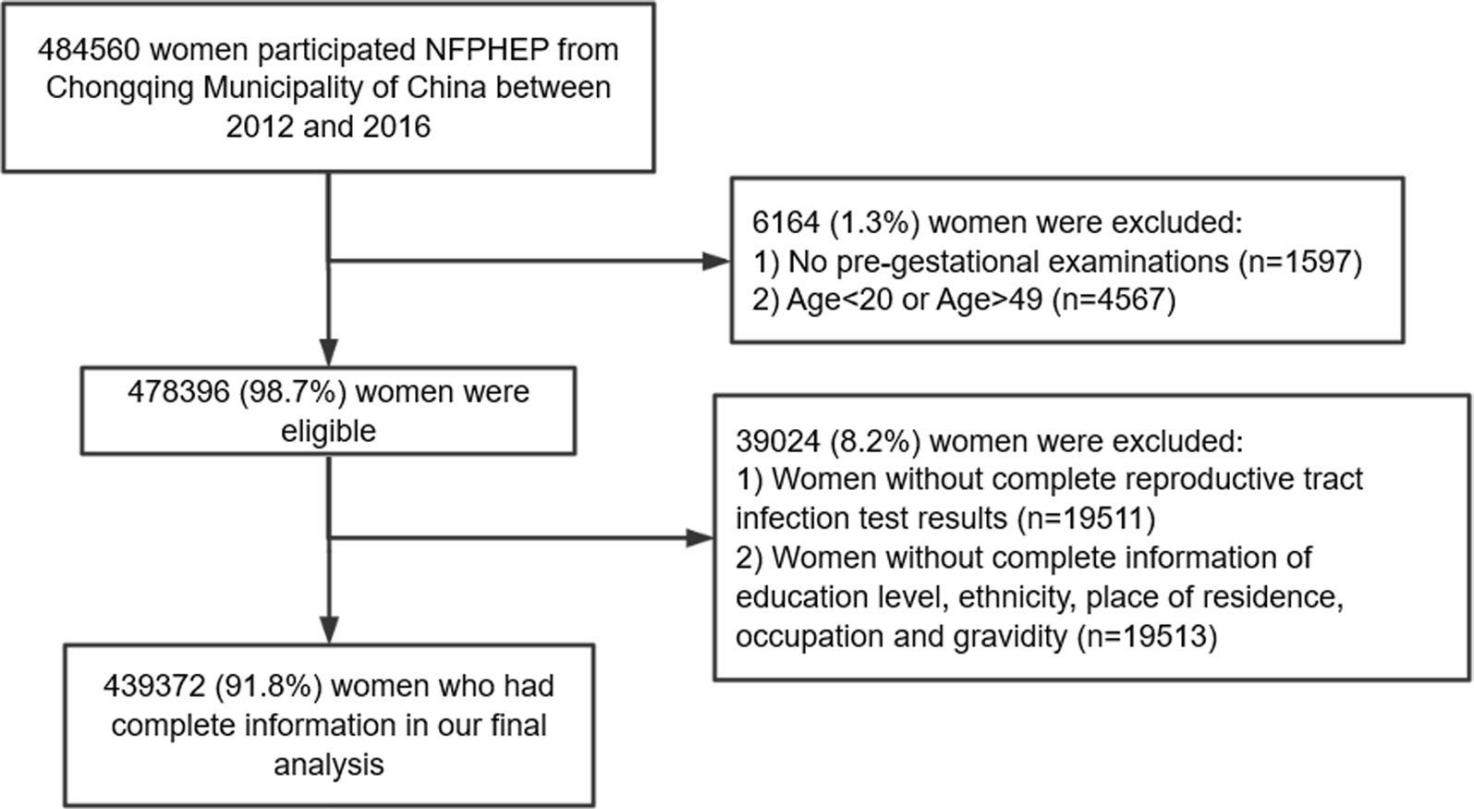
Flowchart of study population

This population-based, cross-sectional study was conducted in Chongqing Municipality of China. The participants were the childbearing women who participated in the National Free Preconception Health Examination Project (NFPHEP). NFPHEP was a national project launched to provide free preconception care, early pregnancy and postpartum follow-up visits for couples who intended to become pregnant within the next 6 months, aiming to reduce birth defects in China. Detailed information about the study design and implementation of the NFPHEP has been described elsewhere previously [14–16]. We extracted Chongqing data from the national NFPHEP database regarding the preconception questionnaire and examination data of 484,560 participants between January 2012 and December 2016. Based on the definition of childbearing women and the age requirement of the Marriage Law in China, 478,396 (98.7%) women between 20 and 49 years old with preconception examinations were eligible for this study. As some of them had incomplete testing for RTIs, our final analyses included 439,372 (91.8%) women, excluding 19,511 (4.1%) subjects with incomplete reports of RTIs status and 19,513 (4.1%) with missing information on basic characteristics, such as age, ethnicity, education level, occupation, place of residence and history of childbearing (Fig. 1). Subjects could participate in the project repeatedly but had to wait for at least 1 year between consecutive rounds.

This study was approved by the Institutional Review Board of the Chinese Association of Maternal and Child Health Studies. Written informed consent was obtained from all participants prior to enrolment.

### Data collection and diagnostic methods

According to the WHO guidelines for RTI care for reproductive health, RTIs can be classified into three categories: (1) sexually transmitted infections (STIs), which are caused by organisms acquired during sexual contact; (2) endogenous infections, which are caused by pathogens usually present within the reproductive tract; and (3) iatrogenic infections, which are introduced through medical procedures [12]. In this study, we focused solely on STIs and endogenous infections.

During preconception care, the demographic and childbearing history of each participant was collected by locally trained health workers using a standardised questionnaire. Blood samples and vaginal and cervical discharges were collected during the preconception health examinations.

In general, during the preconception health examinations, we assessed four types of STIs (*Neisseria gonorrhoeae*, *Chlamydia trachomatis*, *Trichomonas vaginalis* and syphilis) and two types of endogenous infections (bacterial vaginosis and candidiasis) among the participants. The diagnostic methods are listed below:

1. *Neisseria gonorrhoeae*
  Vaginal smear cultures were incubated at 36–37 °C in carbon dioxide for 24–48 h. *N. gonorrhoeae* was identified based on colonial morphology and Gram staining.
2. *Chlamydia trachomatis*
  Golden colloid immunity diffuse test was used to detect antibodies specific for *C. trachomatis*.
3. *Trichomonas vaginalis*
  Microscopic identification of typical motile trichomonas in a wet mount of vaginal discharge.
4. *Syphilis*
  Rapid plasma reagin (RPR) test was used to screen for *Treponema pallidum* (TP) at first among the participants. If the results were positive or equivocal, then a TP particle agglutination assay (TPPA) was used to detect antibodies specific for TP antigens to confirm.
5. *Bacterial vaginosis*
  According to the Amsel criteria, a confirmed diagnosis should meet at least three of the following four criteria: homogeneous white or yellow discharge, the presence of clue cells in vaginal discharge, pH of vaginal fluid > 4.5, and release of a fishy odour after adding a 10% potassium hydroxide (KOH) solution on vaginal discharge [17].
6. *Candidiasis*
  Microscopic identification of fungal hyphae or yeast cells in a wet mount of vaginal discharge.
  These RTIs were all tested in the local laboratories affiliated with medical institutions under qualified quality control mechanisms. The reagent kit specifications were determined by local county laboratories, all of which were approved by the China Food and Drug Administration [18].

### Statistical analysis

Prevalence with its 95% confidence interval (95% CI) was calculated for positive cases in each type of RTI among the participants from 2012 to 2016. Pearson’s chi-square test was used to examine the univariate associations between RTIs and baseline characteristics. Logistic regression was used to evaluate the correlations between the prevalence of RTIs and their related factors among the recruited women. Considering that there might be collinearity between gravidity and parity, adjusted ORs with 95% CIs were calculated after adjusting for age, ethnicity, education level, place of residence, occupation, parity, and history of abortion (both spontaneous and induced).

Factor analysis was used to identify important sociodemographic factors associated with the trend of the RTIs prevalence over the 5 years. Occupation there was classified and assigned by physical labour hours: farmers and workers were class 1; merchants and service staff were class 2; housewives, civil servants and others were class 3. From the seven risk factors that we found in the logistic regression analysis, including age (categorical), education level, occupation, gravidity, parity, history of spontaneous abortion, and history of induced abortion, three common factors were extracted by the analysis. These could explain 70.90% of the original factors. Common factor 1 (could be also called ‘physiology factor’) represented age, gravidity, parity and history of induced abortion; common factor 2 (‘social factor’) represented education level and occupation classification, and common factor 3 (‘disease factor’) reflected the variation in the history of spontaneous abortion. Sensitivity analyses were conducted between the included and eligible groups. All analyses were conducted using the SAS statistical software (9.4 version). A two-tailed level of 0.05 was used to determine statistical significance.

## Results

The average age of the 439,372 women aged 20–49 years who had complete RTI testing results was 28.34 ± 6.29 years. Furthermore, 262,830 (59.82%) of women were educated to middle school level or lower, and 331,306 (75.40%) had an agricultural registered residence. Regarding the history of pregnancy, 310,735 (70.72%) women were multigravida, and 252,541 (57.48%) were multipara.

Among the 439,372 women, 5.03% (95% CI 4.96–5.09%) had at least one definite RTI. The overall positive rate of STIs was 1.45% (95% CI 1.41–1.48%) while endogenous infections had a higher prevalence of 3.69% (95% CI 3.64–3.75%). In addition, 0.11% of participants had both STIs and endogenous infections. As for the six types of RTIs, the positive prevalence of *N. gonorrhoeae* was 0.06% (95% CI 0.05–0.06%); *C. trachomatis*, 0.20% (95% CI 0.18–0.21%); *T. vaginalis*, 0.49% (95% CI 0.47–0.51%); syphilis, 0.73% (95% CI 0.70–0.75%); bacterial vaginosis, 1.28% (95% CI 1.24–1.31%); and candidiasis 2.47% (95% CI 2.42–2.52%) (Additional file 1: Table S1).

The specific prevalence of RTIs in subgroups according to sociodemographic characteristics was shown in Table 1. Women aged 35 years or older had the highest RTI-positive rate (7.58%) among the four age groups, which was nearly two times higher than the rate in women aged 20–24 years (3.72%). Among women with a primary or lower education level, 8.68% of them had at least one RTI, and there was a decreasing trend in RTI prevalence in association with higher education levels. Housewives appeared to have a higher RTI-positive rate (6.38%) than women with other jobs. No significant difference was found in the prevalence of RTIs between different ethnicities (p = 0.297) or places of residence (p = 0.595). As for the history of pregnancy, the prevalence of RTIs was significantly higher in both multigravida (5.74%) and multipara (5.82%) women compared to those who had never conceived (3.31%) or delivered (3.96%). In addition, the burden of RTIs was significantly higher in women with a history of spontaneous abortion (6.63%) or induced abortion (6.16%).

**Table 1:** Prevalence of reproductive tract infections (RTIs) by sociodemographic characteristics among women of childbearing age in Chongqing, China

Characteristic	Total		RTIs present		p-value
	N	%	N	Prevalence (%)	
Overall	439,372		22,098	5.03	
Age, years					< 0.001
20–24	138,520	31.53	5157	3.72	
25–29	155,311	35.35	7086	4.56	
30–34	68,021	15.48	3982	5.85	
35–49	77,520	17.64	5873	7.58	
Ethnicity					0.297
Han	404,983	92.17	20,409	5.04	
Others	34,389	7.83	1689	4.91	
Education					< 0.001
Primary or below	30,997	7.05	2691	8.68	
Middle school	231,833	52.76	11,788	5.08	
High school	90,301	20.55	3864	4.28	
College or above	86,241	19.63	3755	4.35	
Place of residence					0.595
Non-agricultural	108,066	24.6	5402	5.00	
Agricultural	331,306	75.4	16,696	5.04	
Occupation					< 0.001
Farmer	234,538	53.38	12,176	5.19	
Worker	38,794	8.83	1706	4.40	
Merchant	41,892	9.53	1800	4.30	
Service staff	14,920	3.4	710	4.76	
Housewife	24,938	5.68	1592	6.38	
Civil servant	49,613	11.29	2413	4.86	
Others	34,677	7.89	1701	4.91	
Gravidity					< 0.001
0	128,637	29.28	4255	3.31	
≥ 1	310,735	70.72	17,843	5.74	
Parity					< 0.001
0	186,831	42.52	7398	3.96	
≥ 1	252,541	57.48	14,700	5.82	
History of spontaneous abortion					< 0.001
No	422,547	96.17	20,983	4.97	
Yes	16,825	3.83	1115	6.63	
History of induced abortion					< 0.001
No	251,292	57.19	10,520	4.19	
Yes	188,080	42.81	11,578	6.16	

Women’s age, educational level, gravidity, parity, history of spontaneous or induced abortion were associated with RTIs, and after adjusting for other potential factors, there was no association between ethnicity or place of residence and RTIs (Table 2). Compared to women aged 20–24 years, older subjects were more likely to have RTIs, and the relative odds increased in the other three age groups (aOR: 1.17, 1.38, 1.65). The risk of RTIs was significantly higher among women with an education level of primary school or below (aOR: 1.78, 95% CI 1.67–1.90) and middle school (aOR: 1.16, 95% CI 1.10–1.22), compared to the women with college-level education. After considering other factors, gravidity, parity, and history of abortion remained significant factors associated with RTI prevalence.

**Table 2:** The associations of the prevalence of reproductive tract infection with sociodemographic characteristics among women of childbearing age

Characteristic	Crude OR	95% CI	Adjusted OR^a^	95% CI^a^
Age, years				
≤ 24	1.00		1.00	
25–29	1.24	1.19–1.28	1.17	1.12–1.21
30–34	1.61	1.54–1.68	1.38	1.32–1.44
≥ 35	2.12	2.04–2.20	1.65	1.58–1.73
Ethnicity				
Han	1.00		1.00	
Others	0.97	0.93–1.02	0.97	0.92–1.02
Education				
Primary or below	2.09	1.98–2.20	1.78	1.67–1.90
Middle school	1.18	1.13–1.22	1.16	1.10–1.22
High school	0.98	0.94–1.03	1.04	0.98–1.09
College or above	1.00		1.00	
Place of residence				
Non-agricultural	1.00		1.00	
Agricultural	1.01	0.98–1.04	1.00	0.96–1.04
Occupation				
Farmer	1.00		1.00	
Worker	0.84	0.80–0.89	0.94	0.89–1.00
Merchant	0.82	0.78–0.86	1.00	0.95–1.06
Service staff	0.91	0.84–0.99	1.03	0.95–1.12
Housewife	1.25	1.18–1.32	1.29	1.22–1.36
Civil servant	0.93	0.89–0.98	1.20	1.13–1.28
Others	0.94	0.89–0.99	1.09	1.03–1.16
Parity				
0	1.00		1.00	
≥ 1	1.50	1.46–1.54	1.11	1.07–1.15
History of spontaneous abortion				
No	1.00		1.00	
Yes	1.36	1.28–1.45	1.36	1.28–1.45
History of induced abortion				
No	1.00		1.00	
Yes	1.50	1.46–1.54	1.32	1.28–1.35

^a^Adjusted OR with 95% CI were calculated after adjusting age, ethnicity, education level, place of residence, occupation, parity and history of abortion (spontaneous and induced)

**Fig. 2:**
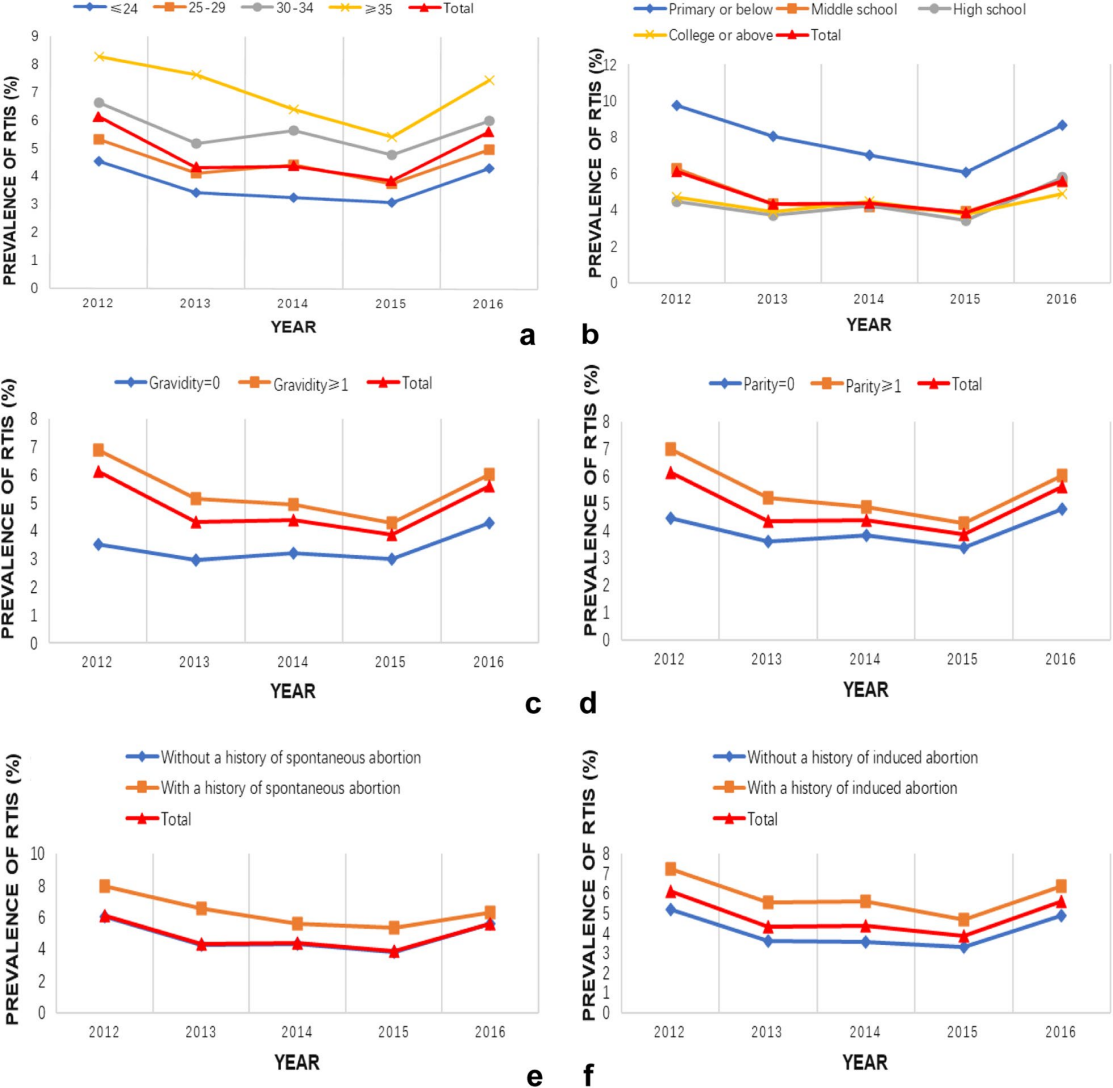
The trend of the prevalence of RTI among childbearing women from 2012 to 2016. The trend of the prevalence of RTI stratified by **a** age, **b** education level, **c** gravidity, **d** parity, **e** history of spontaneous abortion and **f** history of induced abortion

The trends in the overall prevalence of RTIs from 2012 to 2016 were presented in Fig. 2. It was found that the overall prevalence of RTIs decreased from 2012 to 2015, and rose again in 2016 after a consistent fall. The trend was generally consistent across all subgroups stratified by age, education level, or the history of childbearing. Since each subgroup contributed differently to the overall prevalence, we also found that changes in the distribution of these sociodemographic factors over time might have influenced the overall prevalence trend.

**Fig. 3:**
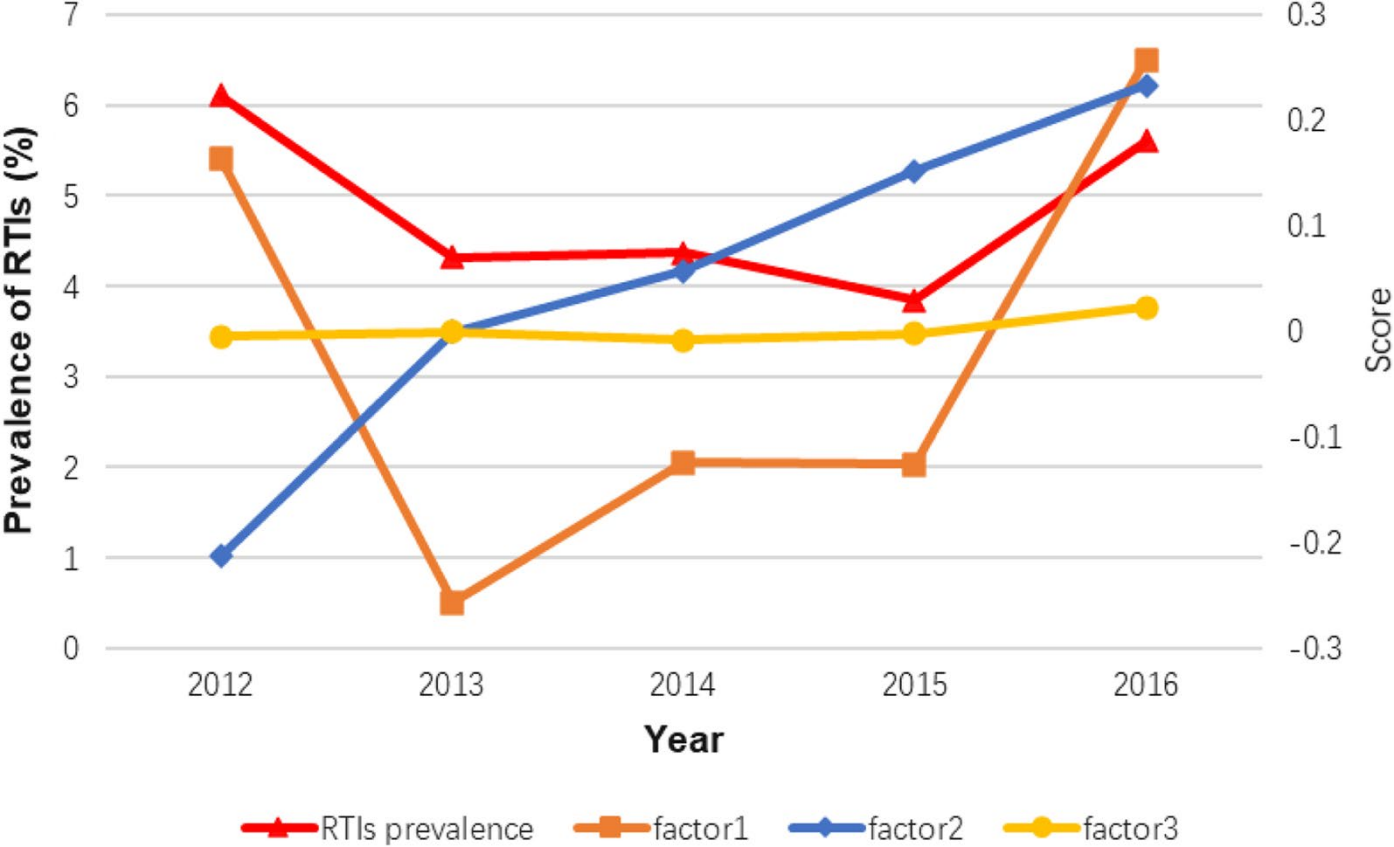
The trend of the prevalence of RTI and average scores of three factors among childbearing women by age from 2012 to 2016. Factor 1 (“physiology factor”) included age, gravidity, parity and history of induced abortion, Factor 2 (“social factor”) included education level and occupation, Factor 3 (“disease factor”) reflected the change of the history of spontaneous abortion

To explore the comprehensive factors that might influence the overall prevalence trend, we presented the trends of the average score of the three common factors extracted from the factor analysis by year (Fig. 3). Our results showed that the trend of factor 1 was most similar to the RTI prevalence trend, while factor 2 might have a negative influence on the prevalence, and factor 3 seemed to have no contribution to the overall trend, which suggested that the ‘battle’ of the changes in ‘physiology factor’ and ‘social factor’ every year might contribute to the final shape of the prevalence trend. Furthermore, the factor ‘history of spontaneous abortion’ might be associated with the rate of RTIs but have no impact on the prevalence trend.

## Discussion

The overall positive rate of RTIs among women of reproductive age was 5.03% in our study. This was much lower than most studies conducted on other specific populations. These studies reported rates such as 30.80% in a cluster sample of 2,000 rural women in the Sichuan province of China [4]; and 58.10% in the stratified cluster sample of 53,652 married women in the rural Anhui Province of China [5]; and 28.30% among general women aged 18–45 years in Goa, India [8]. A 2016 WHO global study found that the prevalence of four types of STIs was as follows: chlamydia (3.8%), gonorrhoea (0.9%), trichomoniasis (5.3%), and syphilis (0.5%) [2]. Unlike the prevalence of other types of RTIs at a relatively lower level, the positive rate of syphilis (0.73%) was high in our study population, and it was also much higher than the national level from 2010 to 2012 in China (0.37%) [18]. However, according to the local study site staff feedback, some of the positive syphilis cases were only based on the RPR test and were not confirmed by TPPA, indicating that our estimated prevalence of syphilis might have been overestimated with a false positive rate of approximately 5–10%, as previous studies reported [19, 20].

These differences might be due to the variations in sample size and the demographic characteristics of the study population. Our study covered women who planned to become pregnant from both rural and urban areas, as the latter may have a greater awareness of reproductive tract diseases and seek pre-pregnancy care. Therefore, lower prevalence was reasonable compared to many studies focusing on rural women or women from disadvantaged social classes only [4, 21, 22]. However, syphilis has a relatively high prevalence, and can cause severe health problems such as infertility, fetal loss, and maternal death [23, 24]. Active prevention and integrated management for syphilis are needed for women of childbearing age in the Chongqing Municipality, China.

In our study, women’s age, education level, occupation, gravidity, parity, and history of abortion were all associated with the presence of RTIs, which is consistent with results from most other studies in China [4, 5, 25]. There were also other factors that might be associated with the prevalence of RTIs but there was lack of information in our study, including RTI-related knowledge, socioeconomic status and condom use [10, 26, 27].

Women with increasing age or reproductive history might have been sexually active for a longer period, thereby increasing their risk of exposure and accounting for the higher RTIs rates [28]. However, this finding was incompatible with that of other studies, which found a higher prevalence of infection among younger women or primigravid women [8, 9]. The difference in legal marriage age might account for this variation. Furthermore, a lower education level might indicate a lack of knowledge regarding the symptoms, severity, and prevention measures of RTIs, leading to lower patient awareness. No significant difference was found in relation to place of residence or ethnicity, indicating no clear disparity in the efficacy of RTI prevention work between women in agricultural and non-agricultural residences, or between Han and other minorities. This might be due to the uniform public health policy and management of health services implemented in the entire Chongqing Municipality.

From 2012 to 2016, the overall prevalence trend of RTIs was V-shaped, with a new peak in 2016 after a steady decrease since 2012. After factor analysis, we found that it was the change in the distribution of the ‘physiology factor’ and the ‘social factor’ over time that might have contributed to this trend, while the proportion of history of spontaneous abortion in participants seemed to have no impact on it. In 2016, the proportion of women with higher age, lower education, and with a pregnancy or delivery history was significantly higher than in that in other years. This could be due to the new Chinese childbearing policy ‘Each couple could have two children in 2016’. As a result, the RTI-positive rate trend might be explained by the change in the proportion of high-risk women from 2012 to 2016.

The most significant strength of our study was its large sample size which covered all 39 counties of the Chongqing Municipality of China. This sample size can provide an accurate estimation of the overall prevalence of RTIs in women of reproductive age in the preconception period. The data contained the information from 2012 to 2016 which can help us to understand RTI trend and its important associated socio-demographic factors. This study has some limitations. Since this was a multicentre study, the reagents and kits used to identify the RTIs were chosen by the local study site. Therefore, their sensitivity and specificity might be different. Second, the data did not include living habits, RTI-related knowledge, or other potential risk factors. Additionally, some confounders, such as the frequency of sexual activity, might have influenced our findings or provided a better explanation for the current results. Moreover, there were differences in the distributions of demographic characteristics between the included and excluded groups (Additional file 1: Table S3), which might have limited bias in the final results.

## Conclusions

In conclusion, the prevalence of RTIs among women of childbearing age who participated in the Chongqing Municipality (China) NFPHEP from 2012 to 2016 was 5.03% and its trend over this time period was V-shaped. Age, education level, gravidity, parity and history of abortion (both induced and spontaneous) were all associated with the prevalence of RTIs. Furthermore, it was the distribution change of the ‘physiology factor’ and ‘social factor’ (including age group, education level, gravidity, parity and history of induced abortion) that contributed to this final trend shape. Pre-conception health education, regular screening and timely treatment of RTIs are needed to prevent the impact of RTIs on maternal health and infant safety. Integrated management and targeted strategies should be developed to deal with the new challenge that is increasingly experienced by elderly and multigravid women who plan to become pregnant under the new Chinese childbearing policy ‘each couple could have two children’.

## Supplementary Information

**Additional file 1: Table S1.** Prevalence of reproductive tract infections among 439,372 women of childbearing age in Chongqing, China. **Table S2.** Prevalence with 95% CI of reproductive tract infections between the included and the eligible group. **Table S3.** The distribution of demographic characteristics between the included and excluded group.

## Data Availability

All data relevant to the study are included in the article or uploaded as Additional file.

## Abbreviations

RTIs: Reproductive tract infections
WHO: World Health Organization
NFPHEP: National Free Preconception Health Examination Project
RPR: Rapid plasma reagin
TP: Treponema pallidum
TPPA: Treponema pallidum particle agglutination assay
95% CI: 95% Confidence interval
