# Supplementary material for: Prevalence of reproductive tract infections among women preparing to conceive in Chongqing, China: trends and risk factors
Source: Reprod Health. 2022 Oct 3;19:197. doi: 10.1186/s12978-022-01502-x (PMC9531418; doi:10.1186/s12978-022-01502-x)
Supplement: Supplementary file 1 — Additional file 1: Table S1. Prevalence of reproductive tract infections among 439,372 women of childbearing age in Chongqing, China. Table S2. Prevalence with 95% CI of reproductive tract infections between the included and the eligible group. Table S3. The distribution of demographic characteristics between the included and excluded group. [file 12978_2022_1502_MOESM1_ESM.docx]

Table S1 Prevalence of reproductive tract infections among 439,372 women of childbearing age in Chongqing, China

(Doc.)

|  | Type of infection | No. of positive cases | Prevalence (95%CI) |
| --- | --- | --- | --- |
| Overall | Any RTIs | 22098 | 5.03 (4.96, 5.09) |
|  | Any STIs | 6362 | 1.45 (1.41, 1.48) |
|  | Any endogenous infections | 16218 | 3.69 (3.64, 3.75) |
|  | Combined STIs and Endogenous infections | 482 | 0.11 (0.10, 0.12) |
| STIs | *Neisseria gonorrhoeae* | 249 | 0.06 (0.05, 0.06) |
|  | *Chlamydia trachomatis* | 868 | 0.20 (0.18, 0.21) |
|  | *Trichomonas vaginalis* | 2134 | 0.49 (0.47, 0.51) |
|  | Syphilis | 3203 | 0.73 (0.70, 0.75) |
| Endogenous infections | Bacterial vaginosis | 5610 | 1.28 (1.24, 1.31) |
|  | Candidiasis | 10848 | 2.47 (2.42, 2.52) |

Table S2 Prevalence with 95%CI of reproductive tract infections between the included and the eligible group

(Doc.)

| **Type of infection** | Included subjects  N=439372 | Eligible subjects  N=478396 |
| --- | --- | --- |
| Any RTIs | 5.03 (4.96, 5.09) | 5.00 (4.94, 5.07) |
| Any STIs | 1.45 (1.41, 1.48) | 1.41 (1.37, 1.44) |
| Any endogenous infections | 3.69 (3.64, 3.75) | 3.67 (3.62, 3.73) |
| Combined STIs and Endogenous infections | 0.11 (0.10, 0.12) | 0.11 (0.10, 0.12) |
|  |  |  |
| *Neisseria gonorrhoeae* | 0.06 (0.05, 0.06) | 0.05 (0.05, 0.06) |
| *Chlamydia trachomatis* | 0.20 (0.18, 0.21) | 0.20 (0.19, 0.22) |
| *Trichomonas vaginalis* | 0.49 (0.47, 0.51) | 0.47 (0.45, 0.49) |
| Syphilis | 0.73 (0.70, 0.75) | 0.70 (0.68, 0.72) |
|  |  |  |
| Bacterial vaginosis | 1.28 (1.24, 1.31) | 1.24 (1.21, 1.27) |
| Candidiasis | 2.47 (2.42, 2.52) | 2.44 (2.40, 2.48) |

Table S3 The distribution of demographic characteristics between the included and excluded group (Doc.)

| **Characteristic** | Included subjects  N=439372 | | Excluded subjects  N=39024 | | | P-value |  |
| --- | --- | --- | --- | --- | --- | --- | --- |
| Age, years |  |  | | <0.001 | | |  |
| 20-24 | 138520 (31.53) | 11911 (30.52) | | |  | | |
| 25-29 | 155311 (35.35) | 15324 (39.27) | | |  | | |
| 30-34 | 68021 (15.48) | 6702 (17.17) | | |  | | |
| 35-49 | 77520 (17.64) | 5087 (13.04) | | |  | | |
| Ethnicity |  |  | | | <0.001 | | |
| Han | 404983 (92.17) | 25965 (85.42) | | |  | | |
| Others | 34389 (7.83) | 4431 (14.58) | | |  | | |
| Education |  |  | | | <0.001 | | |
| Primary or below | 30997 (7.05) | 1416 (7.37) | | |  | | |
| Middle school | 231833 (52.76) | 9402 (48.91) | | |  | | |
| High school | 90301 (20.55) | 3562 (18.53) | | |  | | |
| College or above | 86241 (19.63) | 4842 (25.19) | | |  | | |
| Place of residence |  |  | | | <0.001 | | |
| Non-agricultural | 108066 (24.60) | 12569 (32.21) | | |  | | |
| Agricultural | 331306 (75.40) | 26455 (67.79) | | |  | | |
| Occupation |  |  | | | <0.001 | | |
| Peasant | 234538 (53.38) | 11941 (48.10) | | |  | | |
| Labor worker | 38794 (8.83) | 1916 (7.72) | | |  | | |
| Merchant | 41892 (9.53) | 2216 (8.93) | | |  | | |
| Service staff | 14920 (3.40) | 924 (3.72) | | |  | | |
| Housewife | 24938 (5.68) | 1166 (4.70) | | |  | | |
| Civil servant | 49613 (11.29) | 3995 (16.09) | | |  | | |
| Others | 34677 (7.89) | 2669 (10.75) | | |  | | |
| Gravidity |  |  | | | <0.001 | | |
| 0 | 128637 (29.28) | 9704 (31.84) | | |  | | |
| ≥1 | 310735 (70.72) | 20775 (68.16) | | |  | | |
| Parity |  |  | | | <0.001 | | |
| 0 | 186831 (42.52) | 23045 (59.05) | | |  | | |
| ≥1 | 252541 (57.48) | 15979 (40.95) | | |  | | |
| History of spontaneous abortion |  |  | | | <0.001 | | |
| No | 422547 (96.17) | 37725 (96.67) | | |  | | |
| Yes | 16825 (3.83) | 1299 (3.33) | | |  | | |
| History of induced abortion |  |  | | | <0.001 | | |
| No | 251292 (57.19) | 25567 (65.52) | | |  | | |
| Yes | 188080 (42.81) | 13457 (34.48) | | |  | | |
